# Effects of APOE e4 and Neuropathological Diagnoses on Neuropsychiatric Symptoms: Mediation Analyses and Likely Causation in an Integrated NACC Database

**DOI:** 10.1002/alz.094887

**Published:** 2025-01-09

**Authors:** Terry E. Goldberg, Hyun Kim, Davangere Devanand, Seonjoo Lee

**Affiliations:** ^1^ Columbia University Medical Center, New York, NY USA; ^2^ Columbia Univbersity Medical Center, New York, NY USA; ^3^ Division of Geriatric Psychiatry, New York State Psychiatric Institute, New York, NY USA; ^4^ Columbia University Irving Medical Center, New York, NY USA; ^5^ New York State Psychiatric Institute, New York, NY USA; ^6^ Taub Institute for Research in Alzheimer’s Disease and the Aging Brain, Columbia University, New York, NY USA

## Abstract

**Background:**

Our goal in this study was to identify paths from APOE e4 to neurobehaviors itemized on a neuropsychiatric inventory that involved neuropathologies associated with e4 (amyloid, tau, cerebral amyloid angiopathy, and Lewy bodies) or cognition mediators (memory or global cognitive status), as well as direct paths from e4 to cognition or neurobehaviors.

**Method:**

A total of 1199 cases with available neurobehavioral, cognition and neuropathological data were included. We then conducted a series of causal mediation analyses in R in which e4 always served as the independent variable and Neuropsychiatric Inventory (NPI) neurobehavioral items, when included in the mediation, the outcome. Neuropathologies or cognition served as mediators.

**Result:**

Multiple significant indirect paths from e4 through neuropathologies to neurobehaviors were identified. More refined analyses indicated that neuritic plaques and Braak stage, but not extent of diffuse amyloid plaques, drove the findings. A significant direct effect of e4 to memory was also identified. Additionally, Lewy body disease, when treated as an exposure, had a direct effect on hallucinations in keeping with known features of the disease.

**Conclusion:**

We found strong evidence for partial mediation of NPI symptoms by cognition, suggesting that cognitive limitations that may have influenced understanding (or misunderstanding) the environment with impacts on maladaptive behavior. In addition, neuritic amyloid plaque levels and Braak stage, but not diffuse amyloid plaque extent, were key in NPI mediated associations suggesting the possibility that synaptic failure play an important role in multiple neurobehavioral symptoms in dementia, including psychosis. Last, we found strong evidence that e4 may have direct effects on cognition when we used verbal episodic memory as an outcome, suggesting that medial temporal regions that support memory may be sensitive to non‐amyloidogenic and non‐tau related pathophysiological processes.

